# AVADAR (Auto-Visual AFP Detection and Reporting): demonstration of a novel SMS-based smartphone application to improve acute flaccid paralysis (AFP) surveillance in Nigeria

**DOI:** 10.1186/s12889-018-6187-x

**Published:** 2018-12-13

**Authors:** Faisal M. B. Shuaib, Philip F. Musa, Sisay Tegegne Gashu, Chima Onoka, Salihu Abdullahi Ahmed, Murtala Bagana, Michael Galway, Fiona Braka, Ticha Johnson Muluh, Richard Banda, Godwin Akpan, Ajiboye Tunji, Umar Kabo Idris, Adedolapo Olusoga, Patrick Briand, Nwanyibuife Obiako, Tonia Nebechukwu, Pascal Mkanda

**Affiliations:** 1 National Primary Health Care Agency, Abuja, Nigeria; 2 The University of Alabama at Birmingham, Birmingham, USA; 3 World Health Organization country Representative Office, Abuja, Nigeria; 4 Bill and Melinda Gates Foundation, St. NW, Washington DC, USA; 5 EHealth Africa, Nigeria office, Kano, Abuja Nigeria; 6 Novel-T, Geneva, Switzerland

**Keywords:** Polio, Acute flaccid paralysis, AVADAR, Surveillance, Technology, Smartphone

## Abstract

**Background:**

Eradication of polio requires that the acute flaccid paralysis (AFP) surveillance system is sensitive enough to detect all cases of AFP, and that such cases are promptly reported and investigated by disease surveillance personnel. When individuals, particularly community informants, are unaware of how to properly detect AFP cases or of the appropriate reporting process, they are unable to provide important feedback to the surveillance network within a country.

**Methods:**

We tested a new SMS-based smartphone application (App) that enhances the detection and reporting of AFP cases to improve the quality of AFP surveillance. Nicknamed Auto-Visual AFP Detection and Reporting (AVADAR), the App creates a scenario where the AFP surveillance network is not dependent on a limited number of priority reporting sites. Being installed on the smartphones of multiple health workers (HWs) and community health informants (CHIs) makes the App an integral part of the detection and reporting system.

**Results:**

Results from two phases of tests conducted in Nigeria point to the effectiveness of the App in the surveillance of AFP.

**Conclusion:**

We posit that appropriate use of the App can soon bring about a worldwide eradication of poliomyelitis.

## Background

Typically, polio cases present as acute flaccid paralysis (AFP), which is defined as a sudden onset of paralysis/weakness in any part of the body of a child below 15 years of age [1]. AFP can be caused by a variety of illnesses; it is not limited to polio, and it may be difficult to differentiate polio from non-polio forms of AFP in the early stages. These cases are detected through AFP surveillance, the standard system for polio detection and an effective strategy for case detection. For Nigeria to successfully eradicate this disease, new strategies are needed to complement the existing interventions to detect and report hidden reservoirs of the polio virus early enough for prompt action.

An AFP surveillance network should be sensitive enough to detect all cases of AFP, irrespective of cause, and ensure timely reporting of these cases to relevant health personnel [2]. The World Health Organization (WHO) recommends that a sensitive AFP surveillance network should detect at least one case of non-polio AFP annually per 100,000 children under 15 years of age. In addition, performance indicators for AFP surveillance requires that reporting be timely, complete, and represent the geography and demography of the country [3]. The Nigerian AFP surveillance network is faced with issues of poor detection and reporting of cases in certain areas (e.g., remote and/or security-compromised settlements), therefore increasing the likelihood of hidden reservoirs of the virus.

AFP surveillance is affected by the ability of health workers (HWs) to correctly recognize AFP cases. With the emphasis on polio, many HWs have been inundated with information on its clinical signs and symptoms. This heightened awareness of polio may affect their reporting of AFP cases that they feel are linked to other events. However, a traumatic event such as an intramuscular injection can display weakness or paralysis similar to that caused by the polio virus incubating within the nerve cells. In this case, increased sensitivity for detection of AFP cases would benefit from linking AFP with prior intramuscular injection by asking patients about weakness or paralysis involving injections.

In hard-to-reach areas where routine surveillance is difficult, as is the case in many parts of Nigeria, it is important that all members of the community, not only HWs, are able to detect and report AFP. A further challenge to AFP case detection is that some HWs, and particularly many community informants, do not know the implications of AFP and are therefore unable to accurately identify such cases within their communities. In such a situation, individuals would be unable to provide valuable information that would otherwise lead to case detection during routine AFP surveillance activities by assigned surveillance personnel, also known as Disease Surveillance Notification Officers (DSNOs), in Nigeria.

Even when cases are reported, the bureaucracy in reporting or the delay when the DSNO does not respond rapidly may discourage HWs and community informants from actively taking part in the detection of cases. This results in further disruption of effective surveillance for AFP. Poor reporting methods for AFP cases also affect the sensitivity of the surveillance network.

The eradication of poliomyelitis has been a global health priority since the 41st World Health Assembly in 1988 [4]. Since then, huge amounts of resources (financial, manpower, logistics, etc.) have been mobilized globally and within countries to achieve this goal. As a result, polio came close to being eradicated across the world except for three countries considered endemic for the disease: Afghanistan, Pakistan, and Nigeria [2]. Nigeria was declared polio free in 2015 [5]. However, due to the insurgency that started in 2009, certain parts of the country including Borno state were mostly inaccessible to healthcare workers. The result was that, unknowingly, the polio virus was still being harbored. This was before the Auto-Visual AFP Detection and Reporting (AVADAR) smartphone application (App) was developed. In 2016, three new cases were detected in Borno state [4]. To increase AFP case detection rates, innovations must address challenges to the surveillance system and improve reporting in areas where: 1) AFP surveillance key indicators are not being met; 2) AFP surveillance is not being performed systematically; and 3) access to communities is a challenge.

### New SMS-based technology to improve AFP surveillance

Studies have shown that the use of mobile technology for health in developing countries is an innovative and cost-effective way of reaching populations, given the exponential rise in the use of mobile phones [6]. The increase in bandwidths and falling cost of smartphones has enabled more people to own smartphones.

The use of smartphones is revolutionizing healthcare globally. Smartphones are used for training of healthcare workers and are invaluable in communication and collaboration between health services [7]. Developing countries are not being left behind in the use of smartphones in healthcare. For example, they are used in India for the diagnosis and management of chronic diseases such as diabetes, HIV, and hepatitis [7].

AVADAR was designed to address problems with detection and reporting of AFP cases, and therefore improving the quality of AFP surveillance by supporting: 1) weekly reporting by HWs and community health informants (CHIs) on the presence or absence of AFP cases in their respective areas; and 2) instant notification of an AFP case with a minimal set of information being collected and sent directly to the DSNO responsible for the area (district or Local Government Area (LGA)).

AVADAR was conceptualized by Dr. Faisal Shuaib (the first author) and developed by collaborative efforts of multiple developmental partners including The Bill and Melinda Gates Foundation (BMGF), WHO, and Novel-T (a Swiss-based software company). The partners also collaborated to ensure all study activities and requirements were achieved as needed, from project conception and implementation to post-implementation monitoring. The App creates a scenario where the AFP surveillance network is not dependent on a limited number of priority reporting sites. It is installed on the phones of a large number of HWs and CHIs, potentially making every health facility, health worker, and community-based AFP volunteer part of the detection and reporting system.

This two-stage study was conducted to determine a proof of concept and the feasibility of a new smartphone App, AVADAR, to enhance polio detection and eradication. Conducted across a wide range of communities between June and August 2016, the pilot study included 198 participants in two LGAs in Kwara state and Kuje area council in the federal capital territory of Nigeria. We subsequently scaled up the study in 2017 to cover eight LGAs in Borno state (in northeastern Nigeria). The App was installed on mobile devices provided to participants during the 8-week pilot study and for the scaled-up study in Borno state.

In the pilot study, all wards in the Oyun LGA in Kwara state met the set target of 80% for timeliness and completeness of reports. Four out of the ten wards in the Kuje area council met the 80% target for completeness, while none of the wards met the target for timeliness of reporting. In both Oyun and Kuje, there was 83% documented reporting of true AFP by wards, compared with 36% during the pre-pilot period; this is an improvement of over 130%. The App demonstrated an improvement in AFP reporting by tackling issues with the surveillance system on a small scale.

The potential for the App to enhance AFP surveillance and the possibility for its use in surveillance of other priority diseases for public health necessitated study on an even larger scale to gain a better idea of its impact on a sample more representative of the Nigerian AFP surveillance system. This is why it was further expanded to Borno state.

## Methods

### Pilot study setting

Kuje is one of the six area councils that make up Nigeria’s federal capital territory. The Kuje area council is comprised of ten wards spread across an area of 1644 km^2^. Oyun is one of the 16 LGAs of Kwara state. It is comprised of eleven wards spread across an area of 476 km^2^. Kuje and Oyun are both located in the north central geopolitical zone of Nigeria along with five other states (Benue, Niger, Kogi, Nasarawa, and Plateau).

Kuje Area Council, with an estimated population of 112,777 under 15 years of age, has good AFP surveillance indicators and a good cellular communication network. On the other hand, Oyun LGA has an estimated population of 60,294 under 15 years of age, has poor AFP surveillance indicators, and a limited cellular communication network. To provide a mix of contexts for the pilot study, these two LGAs were chosen using the following criteria: AFP surveillance performance indicators, cellular network quality, and population size. Kuje area council (federal capital territory) was selected as the urban site while Oyun LGA (Kwara state) was selected as the rural site for the study. Both LGAs possess a large enough population to ensure that there is an expectation of at least two AFP cases reported annually. Within these LGAs, functional government-owned health facilities, private health facilities (with high patient turnover), and some literate community informants linked to these health facilities were enrolled in the pilot study.

Participants were drawn from the existing surveillance network of HWs and CHIs. However, more CHIs were added to ensure an even geographical spread across the LGAs. To make the AVADAR system more sensitive to detect AFP cases, we focused on influential people from the communities that had a higher likelihood of coming across AFP cases. Table 1 shows the classification of HWs and CHIs by their roles in the existing public health system. The pilot study ran for a period of 8 weeks in each location. The Kuje study was conducted from weeks 33 to 40, while the Oyun pilot study ran from weeks 35 to 42 in 2016.

**Table 1:** Distribution of AVADAR pilot participants by ward in Kuje area council

Wards	Number of health workers	Number of community informants	Total	%
Chibiri	7	5	12	11%
Gaube	12	8	20	18%
Gudun Karya	2	9	11	10%
Gwargwada	1	4	5	4%
Kabi	3	6	9	8%
Kuje	7	16	23	21%
Kujekwa	2	5	7	6%
Kwaku	3	5	8	7%
Rubochi	4	4	8	7%
Yenche	1	8	9	8%
Total	42	70	112	100%

The pilot study was conducted with the help of two main groups: 1) HWs made up of midwives, nurses, and surveillance focal persons; and 2) CHIs comprising traditional birth attendants, traditional spiritual healers, traditional bone setters, influential youths, village heads, patent medicine vendors, and traditional barbers.

### Training HWs and CHIs on the use of AVADAR

HWs and CHIs were trained in small groups of no more than 20 people by facilitators at the LGA level. A mixture of practical demonstrations and role play sessions were conducted for informants to learn how to effectively use the AVADAR App for case reporting. At the end of the training, relevant HW and CI information was collected (e.g., name, phone number, and health facility) and uploaded to the server which connects each HW and CHI to their respective DSNO. Once a HW or CHI reports a suspected case, the server immediately sends an SMS notification with case details to the DSNO’s phone for further investigation.

All informants who agreed to participate in the pilot study (and the subsequent scaled-up study in Borno state) were provided with mobile phones with the AVADAR App pre-installed. The AVADAR App contains an embedded 30-s video of a child with symptoms of AFP, and a case investigation form that HWs and CHIs use to record case details of each child suspected of having AFP. The video is used as a learning tool to increase the ability of informants at community level to recognize the signs and symptoms of AFP for timely case detection. At a designated day and time (e.g., Monday, 11 am) each week, the video appeared on the phone of study participants.

**Fig. 1:**
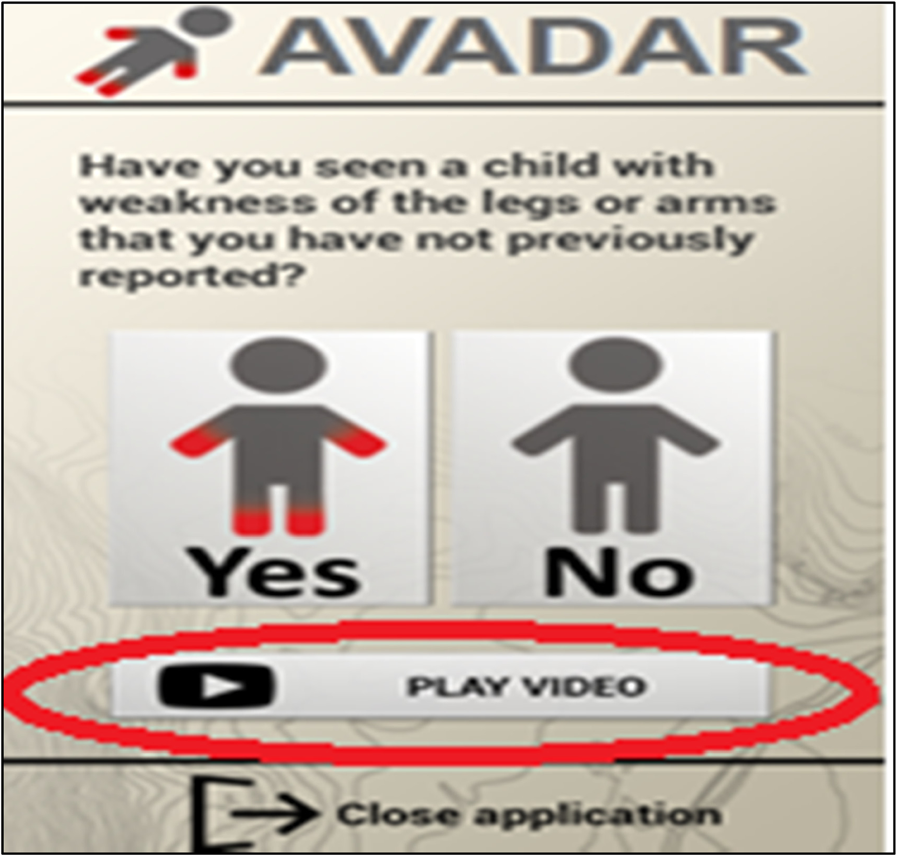
AVADAR dialog box for health workers

Shown on the video is a child who is unable to stand on his/her lower limbs or has some weakness while crawling. After the brief video, a dialogue box pops up that asks the question: “Have you seen a child with weakness of the legs or arms that you have not previously reported?” After this question, two boxes appear asking the health worker to press “Yes” or “No” (Fig. 1).

If the answer is “No” a text message is sent to a central server noting that in the previous 1 week the HW whose cell phone number is already registered has not seen an AFP case.

However, if the answer is “Yes”, other questions would follow:

- When did the weakness occur? (About 60 days or less/More than 60 days ago)
- Please enter child’s name (First and Surname)
- Please enter the parent’s name (First and Surname)
- Please enter the parent’s phone number
- Please enter the child’s address
- Are you at the child’s place of residence?
- Please record your GPS coordinates if you are currently near the child’s place of residence
- Please confirm the date and submit your “Presence of AFP Report” by pressing the button below.

Informants in the study reported suspected AFP cases on a weekly basis to a cloud-based server using the AVADAR application on the mobile phone provided. Where a suspected AFP case is not found, a “zero report” was sent at the end of the week. Once a HW or CHI sends a suspected AFP case report, the server automatically sends a notification alert to the respective DSNO’s mobile phone with a summary of the suspected AFP case information (child’s name, date of onset of symptoms, geographic coordinates of child’s house address, etc.). This prompts an investigation into whether the suspected AFP case is true or not.

### AVADAR functionality, notification, and feedback systems

The DSNOs use a customized open data kit (ODK) application form on their mobile phones to investigate all reported AFP cases and send confirmed case details to the AVADAR server. Data is collated from the AVADAR servers and used for data analysis.

**Fig. 2:**
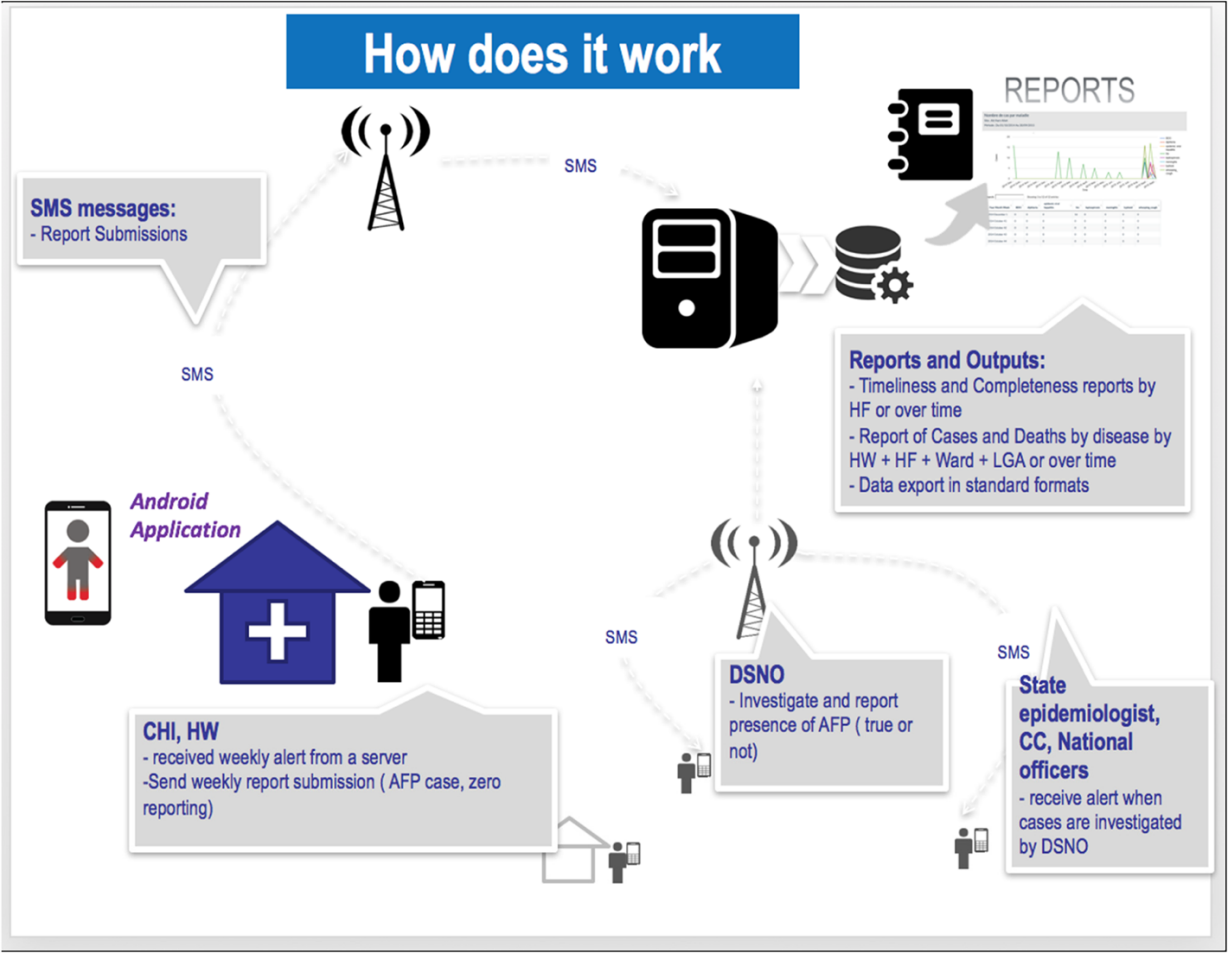
AVADAR notification and feedback loop systems. AFP acute flaccid paralysis, cluster consultants (CC), CHI community health informant, DSNO Disease Surveillance Notification Officer, health facility (HF), HW health worker, LGA Local Government Area, SMS short message service

As depicted in Fig. 2, once a health worker presses the “send” button, a text is sent to a central server which triggers a communication SMS to be sent to the DSNO to investigate. It is easy for the central server to determine where the case is coming from because the database is pre-configured with cell phone numbers of the HWs and CHIs, which health facility they work in, and which ward or LGA they live in. Therefore, in every case, it is clear which ward/district the notification is coming from. Therefore, this does not require that the health facility is geocoded. However, if a health facility is geocoded, then the information can be further linked with maps to project the exact location of the health facility or community from where the notification came.

In order to ensure follow-up of the investigated AFP cases, the alert goes to multiple health personnel at different levels to monitor the outcome of the investigation. The state epidemiologist, the WHO state surveillance officer, and the LGA facilitator will get the same alerts, and the feedback loop from the DSNO to these individuals (and the health worker) will indicate the outcome of the investigation.

### Troubleshooting support and field supervisory visits

After training and distribution of mobile devices to informants, a HelpDesk phone line was provided for informants to call if they needed troubleshooting assistance to address minor technical issues encountered while using the phones or AVADAR App (e.g., incorrect date/time phone setting, uninstalled AVADAR application on the phones, etc.). In addition to this, joint partner field monitoring and supervision visits were conducted on a weekly basis by WHO and eHA to identify challenges or issues impeding timely reporting by informants, to resolve technical issues that could not be handled via phone, to provide brief capacity-building sessions in the field, and to ensure timely investigation by DSNOs when case alerts were reported.

## Results

Participants in the study comprised health workers and community informants from all wards in the pilot LGAs. In Kuje area council, there were 112 participants, while 86 participants took part in the study in Oyun LGA. In both LGAs, there were more community informants than health workers participating in the study, but an equal number of community informants were selected for both locations. The details are shown in Tables 1 and 2.

**Table 2:** Distribution of AVADAR pilot participants by ward in Oyun Local Government Area, Kwara state

Wards	Number of health workers	Number of community informants	Total	%
Erin North	3	7	10	12%
Erin South	1	7	8	9%
Igosun	1	7	8	9%
Ijagbo	1	7	8	9%
Igbonna	3	6	9	10%
Ikotun	3	6	9	10%
Ipee	1	8	9	10%
Irra	2	6	8	9%
Ojoku	1	6	7	8%
Aho	0	6	6	7%
Ilemona	0	4	4	5%
Total	16	70	86	100%

The performance of HWs and CHIs in the AVADAR surveillance network was measured on two primary indicators: completeness and timeliness. “Completeness” refers to when HWs or CHIs send in at least one report (AFP or “zero” report) within a week. “Timeliness” refers to when HWs or CHIs send in at least one report (AFP or “zero” report) within 48 h of receiving the video notification reminder on their mobile devices. All wards in Oyun LGA met the set target of 80% for timeliness and completeness of reports, respectively. In Kuje area council, four out of the ten wards met the 80% target for completeness while none of the wards met the target for timeliness of report. Kuje LGA had a lower proportion of reports sent by participants (92%) compared with Oyun LGA (99%). However, all reports (100%) forwarded to the server in both LGAs were investigated by the DSNO within 48 h of notification in the respective locations.

Overall, in both LGAs, 83% reporting by wards of true AFP cases was documented during the AVADAR study compared with 36% using the traditional AFP surveillance system during the pre-pilot period. In Oyun LGA, 28 suspected AFP cases were reported, out of which seven (or 25%) were confirmed to be true after investigation. In Kuje area council, 40 AFP cases were reported with 20 (or 50%) confirmed to be true cases. In Kuje LGA, 90% of notified AFP cases were within 14 days of onset of paralysis (which is the optimal timeframe) compared with 100% in Oyun LGA. During the 8-week study period, both LGAs (Kuje and Oyun) reported a higher number of cases compared with other LGAs with a comparable population of those aged under 15 years.

Upon completion of the pilot study, the AVADAR application was then scaled up to eight LGAs in Borno in November 2016, with 527 HWs and CHIs added into the AVADAR network (Table 3).

**Table 3:** Distribution of AVADAR participants by Local Government Areas (LGAs) in Borno state

LGAs	Number of health workers	Number of community informants	Total	%
AskiraUba	18	12	30	6%
Biu	8	40	48	9%
Chibok	13	31	44	8%
Gubio	6	20	26	5%
Jere	23	124	147	28%
Kaga	2	20	22	4%
Konduga	11	49	60	11%
Maiduguri Municipal Council	17	133	150	29%
Total	98	429	527	100%

**Fig. 3:**
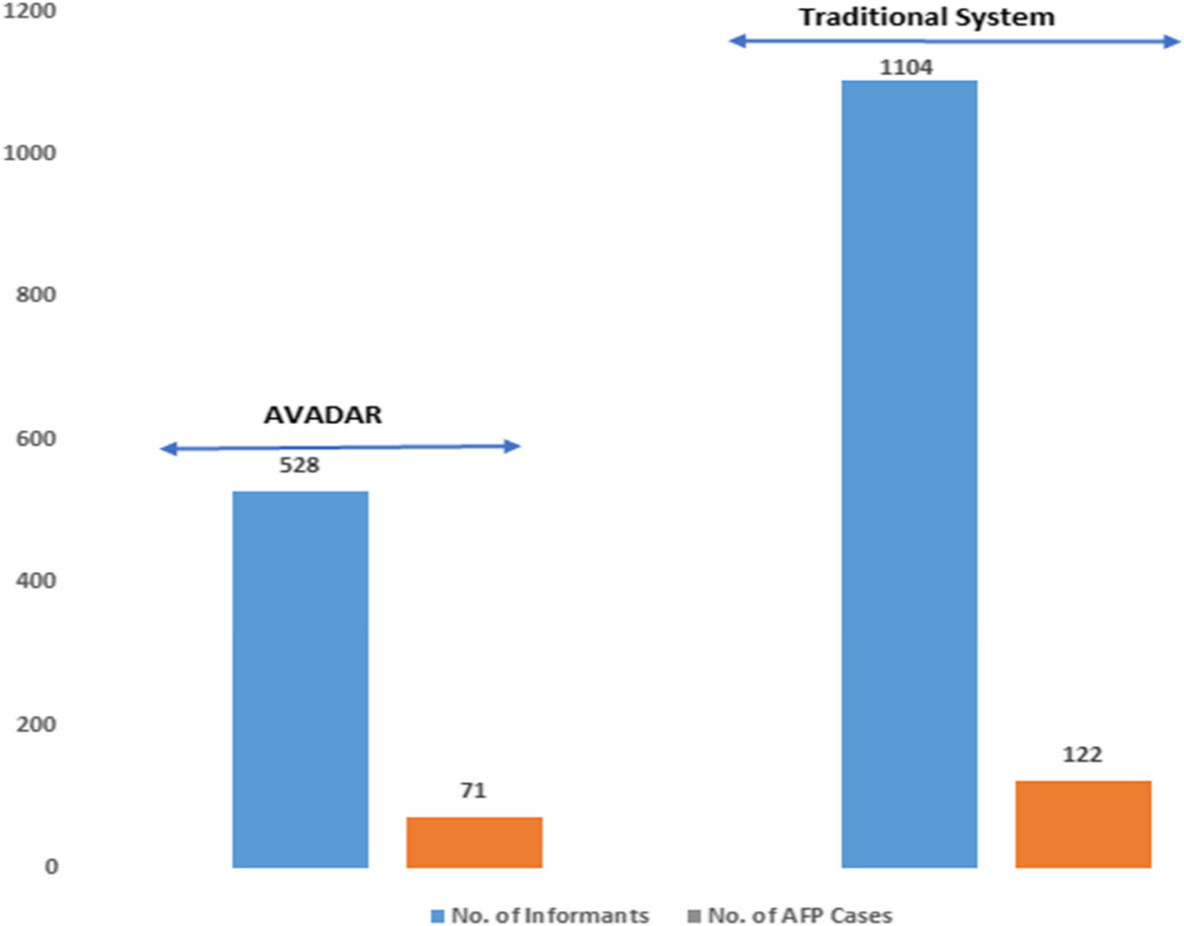
AFP cases reported through AVADAR versus the traditional system across eight LGAs in Borno state (week 48 in 2016 to week 35 in 2017). AFP acute flaccid paralysis

There were 193 AFP cases reported in Borno state between week 48 in 2016 and week 35 in 2017. Of these, 122 cases were reported by the traditional surveillance system while an additional 71 were detected via AVADAR. During the scaled-up study period, there were only 528 informants in the AVADAR network while the traditional system had 1104 informants (almost double the AVADAR personnel). While traditional system detected more cases (shown in Fig. 3), the AVADAR system performed better when the analysis was assessed per capita. For every 100 informants, an average of 13 AFP cases was reported through the AVADAR system while only 11 AFP cases were reported through the traditional system.

We also performed a similar comparison of the number of cases detected via AVADAR versus the traditional surveillance system in security-compromised wards in Borno state. Of the 13 security-compromised wards within the state, AVADAR reported 24 of the AFP cases (or 68.6% of the total) compared with the traditional surveillance system (only 11 cases or 31.4% of the total).

In three previously silent wards (i.e., where no AFP cases were detected or reported in 2016), the AVADAR system was sensitive enough to detect one AFP case in each ward within Borno state (Table 4). The three previously silent wards were Mussa, Mandaragirau, and Mboakwa. It should be noted that the only AFP case reported in Mussa ward was through AVADAR.

**Table 4:** Acute flaccid paralysis (AFP) reported from silent wards in Borno state through AVADAR versus the traditional system (week 48 in 2016 to week 35 in 2017)

S/N	LGA	Silent wards (January–December 2016)	AFP reported by AVADAR	AFP reported by traditional system	Total AFP
1	Askira/Uba	Mussa	1	0	1
2	Biu	Mandaragirau	1	2	3
3	Chibok	Mboakwa	1	2	3
	Total		3	4	7

Local Government Area, Serial Number (S/N)

## Discussion

The results of the pilot study and subsequent scaled-up study in Borno state showed an increase in the number of AFP cases detected and reported following the use of the AVADAR application. They showed its potential to detect cases in security-compromised and silent reporting areas. This supports the concept of the App. When a vivid video of acute flaccid paralysis is utilized as an educational reminder tool for HWs and CHIs, and routine supportive supervision field visits are conducted to resolve technical phone issues/facilitate capacity retraining sessions for informants, there would be an increase in the effective detection and timely reporting of AFP cases.

In comparison with the traditional surveillance system, the use of AVADAR led to a considerable increase in the number of AFP cases detected and reported. The number of AFP cases reported during the 8-week pilot study period was more than twice the number of cases detected via the traditional surveillance system during the 33-week period prior to the start of the pilot study in Kuje and Oyun. Furthermore, the Borno state study showed that AVADAR had a larger per capita AFP case detection rate than the traditional surveillance system.

The AVADAR video educational tool resulted in an increased awareness level of AFP amongst community informants compared with the traditional system. Among healthcare workers, there are varying degrees of knowledge regarding the definition of AFP. A recent study in Kano state (in north central Nigeria) showed that DSNOs and focal persons in reporting sites had the highest level of knowledge of the standard case definition compared with clinicians and other health workers such as community health extension workers (CHEWs) [8]. Since these health workers are responsible for identifying AFP cases in the community, this may potentially affect the accuracy of AFP case reporting. With the AVADAR system, the emphasis shifts from polio to include all potential AFP cases, irrespective of cause. This provides major benefits for public health, especially in developing countries.

Compared with the traditional surveillance system, AVADAR showed greater potential to detect more AFP cases in security-compromised areas. AFP case detection in security-compromised LGAs was three times higher through AVADAR than through the traditional system. Across silent reporting wards in Borno state, AVADAR was sensitive enough to detect AFP cases in locations that had not received any AFP case report by the traditional system within the entire 2016 period. In this final push to eradicate polio from Nigeria, all resources are needed to ensure that the surveillance network is sensitive enough to detect hidden reservoirs of the virus. Using AVADAR, we would be less likely to prematurely declare that polio is eradicated.

Although AVADAR led to an increase in the number of AFP cases detected and reported, not all target metrics were met for the indicators of completeness and timeliness. For the pilot study, Oyun LGA met both timeliness and completeness targets during the 8-week study. However, Kuje area council was only able to achieve 80% target for completeness in four wards. Kuje had consistently poor network connectivity, which may have caused some SMS reports from informants to be delayed or not get through to the server. Kuje also had a larger network of participants responsible for sending weekly case reports compared with Oyun. For Borno state, all LGAs achieved over 70% completeness and timeliness. However, none met the 80% target for completeness and timeliness. This was also due to poor network accessibility in most LGAs within Borno state, coupled with the fact that it is a much larger area (over four times that of the pilot study LGAs).

Ensuring that reporting of AFP cases is not hampered by bureaucracy or inefficiency on the part of surveillance officers is important in making the process more acceptable and appealing, especially to members of the community. In a previous study [9], some 38.5% of respondents who had seen AFP cases in the preceding 3 years took no action on the cases. The advice they gave to 29.5% of the victims’ parents was to go to their nearest health facility. Unfortunately, only 14.0% reported the case to a health worker. Having the AVADAR App installed on the mobile phones of community members provides an easy and efficient process for prompt reporting of cases.

Despite the very positive results from this study, there were challenges encountered during the pilot study and implementation phase in Borno state. The challenges included poor network connectivity in remote locations, insurgent attacks in security-compromised locations, and missing/stolen informant phone devices. Mitigation strategies were employed to resolve these. Examples included transitioning informant phone lines to alternative network providers available in the locations, intensifying efforts by eHA technical officers to resolve most troubleshooting issues via the HelpDesk line, joint partnering of field visits (by WHO, Ministry of Health, eHA) with military personnel when available, and buffering equipment stock available for replacement of missing or stolen phones.

### Large scale implementations

When planning scaled-up efforts beyond Borno state, there are aspects of AVADAR that must be considered before it can be approved for expansion to additional LGAs or countries. These include: the status of surveillance performance indicators, proportion of displaced and nomadic populations, security accessibility, network availability, ample equipment buffer stock supply for replacement purposes, and geographic spread of proposed LGAs/districts. In addition, the cost-effectiveness and sustainability of the utilization of smartphones should be considered.

The inclusion of a focus group discussion with the study participants in the study protocol would shed more light on the challenges and areas to focus on during the pre-implementation phase of expansion efforts. Quantitative data alone would not be sufficient. To enhance the effectiveness of the study, various cultural and local factors were taken into account and applied. While the results clearly show that the use of AVADAR led to more cases being reported, it is important to identify the differences in the detection and reporting of cases by health workers compared with community members. This would indicate specifically to whom the App could be more tailored and whether it would be an optimal and cost-effective intervention for further use and expansion to additional geographic locations in the polio eradication campaign.

## Conclusions

The AVADAR App demonstrates an ability to improve AFP reporting by tackling issues with the surveillance system on a small scale. The study findings show that AVADAR, in conjunction with health education and sensitization, are not only useful for health workers but also invaluable in engaging community members to report cases of AFP through a community-based surveillance approach. The promise it holds for AFP surveillance and the possibility for its use in surveillance of other diseases necessitates its study on a larger scale to get a better idea of its impact on a sample more representative of the Nigerian AFP surveillance system, as well as that of other polio high risk countries.

## Abbreviations

AFP: Acute flaccid paralysis
AVADAR: Auto-Visual AFP Detection and Reporting
BMGF: Bill and Melinda Gates Foundation
CHI: Community health informant
DSNO: Disease Surveillance Notification Officer
eHA: EHealth Africa
HW: Health worker
LGA: Local Government Area
ODK: Open data kit
SMS: Short message service
WHO: World Health Organization
